# Neolactoferrin As a Stimulator of Innate and Adaptive Immunity

**Published:** 2013

**Authors:** A.D. Chernousov, M.F. Nikonova, N.I. Sharova, A.N. Mitin, M.M. Litvina, P.E. Sadchikov, I.L. Goldman, A.A. Yarilin, E.R. Sadchikova

**Affiliations:** Institute of Immunology, Federal Medical and Biological Agency, Kashirskoe shosse, 24/2, Moscow, Russia, 115478; Institute of Gene Biology, Russian Academy of Sciences, Vavilova Str., 34/5, Moscow, Russia, 119334

**Keywords:** Recombinant human lactoferrin, Neolactoferrin, immunity, inflammation, cytokines, transcription factors

## Abstract

The effect of the innovative product Neolactoferrin, a natural combination of recombinant human lactoferrin (90%) and goat lactoferrin (10%) isolated from the milk of transgenic goats carrying the full-length human lactoferrin gene, on human immune system cells was studied. Neolactoferrin enhanced the production of IL-1β. Neolactoferrin saturated with iron ions increased the synthesis of pro-inflammatory cytokine TNFα. It determined the direction of the differentiation of precursor dendrite cells. Under the action of T cells, Neolactoferrin amplified the expression of the transcription factors responsible for the differentiation of Th- and Treg-cells and stimulated the production of both IFNγ and IL-4. The results suggest that Neolactoferrin exhibits an immunotropic activity and hinders the development of immune inflammatory processes. Iron saturation of Neolactoferrin increases its pro-inflammatory activity.

## INTRODUCTION

Lactoferrin (LF) is the key bactericidal protein in human milk that protects neonates against infections. LF exhibits antimicrobial [1-3], antiviral [4-6], and antifungal [7, 8] activities. LF has also been shown to affect the antibiotic-resistant microflora, while microorganisms can manage no genetic adaptation to it [9, 10]. When used together with antibiotics, LF enhances their effect [11, 12]. Despite its strong antimicrobial properties, LF does not suppress the vital activity of the normal microflora of the gastrointestinal tract [13, 14]. Furthermore, it stimulates the growth of bifidobacteria by supplying the iron ions required to ensure their vital activity [15]. The other biological activities of LF include immunomodulation [9, 16], antioxidation [17, 18], and anti-inflammatory [19] activity. LF and its derivatives (lactoferricins) have been found to suppress the progression of tumors and metastases in experimental animals [20-22].

The mechanism behind the biological activities of LF has been well studied [23-25]. The bactericidal effect of LF was found to be caused both by its direct action on pathogenic microorganisms and by its ability to activate the immune system of the organism via the stimulation of innate immunity, as well as activation and differentiation of immune-competent cells [26]. Researchers endeavored to isolate pure human lactoferrin (hLF) in an attempt to use it as a component of functional feed products or various biologically safe new-generation drugs. Researchers at the Herzen Moscow Oncological Research Institute have verified the feasibility of using hLF in such a way: they used hLF isolated from human breast milk to design high-efficiency drugs with a broad therapeutic effect [27-30], including injection forms [1]. Unfortunately, the demand for hLF cannot be met because of the problems associated with breast milk supply.

Lactoferrin isolated from bovine milk (bovine lactoferrin, bLF) with biological activity largely similar to that of hLF has been widely used over the past decade [32, 33]. However, despite the success in using bLF [34], a decision was made to use recombinant human lactoferrin (rhLF) instead of the “alien” bLF as it is done for some other biologically active animal proteins. There is only 67% homology between the amino acid sequences of hLF and bLF [35]. The differences in the primary structure cause differences in the secondary and tertiary structures of these proteins, which may determine their functional features. Certain differences in the structure of hLF and bLF in various human organs and tissues have already been revealed [36]. Thus, the receptor of small intestine cells was found to show higher specificity to hLF than to bLF; this difference can be to a significant extent attributed to the hLF structure [37]. The hLF receptor is believed to participate in iron absorption in the small intestine in humans [38]. Iron is typically transported through the apical membrane of the small intestine by the divalent metal transporter-1 (DMT-1). Iron bound to hLF cannot penetrate into the cell via DMT-1; the hLF receptor performs that function. Once hLF is inside the cell, it binds to the nucleus, where it is believed to act as a transcription factor and induce the biosynthesis of signaling proteins, such as caspase-1 and interleukin-18. These proteins subsequently enter circulation as a systemic signal. This pathway is considered to be the minor one; only ~10% of hLF is transported via this pathway. The main pathway of hLF penetration into epithelial cells results in the degradation of ~90% of the protein and iron release.

hLF receptors similar to the small intestinal receptor have been found in salivary glands, the heart, skeletal muscles, adrenal glands, and the pancreas [39]. Two other types of receptors were detected in the liver: the low-density lipoprotein receptor-related protein (LFP) and the asialoglycoprotein receptor (ASGPR).

Degradation of bLF and hLF yields the so-called lactoferricins denoted by the symbols B [40] and H [41], respectively. These lactoferricins differ in terms of both the amino acid sequence and their biological activity.

Immunologists believe that full biological safety of bLF for humans can be ensured only if this protein is used as a component of food products, whereas hLF can also be used as a component of the injection form of drugs.

rhLF has been produced in different countries by modern bioengineering methods using plants [42, 43], microscopic fungi [44], and animals [45, 46] as producers.

In Russia, rhLF has been produced as a component of goat milk within the framework of the Belarus–Russia Union State program [47]. Its physicochemical parameters and biological activity correspond to those of natural hLF [48, 49]. This protein was used to produce an innovative product, Neolactoferrin (Neolact), a combination of rhLF and goat lactoferrin (gLF) in transgenic goat milk at a 90 : 10 rhLF : gLF ratio.

Goat lactoferrin was experimentally found to enhance the expression of the *NF-κB *gene and synthesis of the tumor necrosis factor (TN Fα), which is extremely important for the activation of innate immunity; however, it has no effect on the activation of interleukin-1 (IL-1) synthesis.

This study is focused on the joint effect of rhLF and gLF on innate immunity indicators in humans. The ability of Neolact with different iron contents (4% (Fe-) and 16% (Fe+)) to induce innate immunity, to enhance the presentation capacity of dendritic cells, to determine the direction of differentiation of T-cell precursors, and to boost the synthesis of major adaptive immune response cytokines (interferon-γ (IFNγ) and IL-4) was studied.

## EXPERIMENTAL

The activity of Neolact samples was assessed in a concentration range from 0.1 to 100 μg/ml under incubation with the tested cells for 18 h at 37°C.

Mononuclear cells (mostly lymphocytes) were isolated from human whole blood via centrifugation using the one-step ficoll-verographin density gradient (density of 1.077 g/ml). The fraction was obtained by incubating blood mononuclear cells in 24-well plates (Costar, USA) for 1 h at 37°C.

The human dendritic cell line HTSC.IL-10 was cultured and stored at the Lymphocyte Differentiation Laboratory (Institute of Immunology, Russia) [50].

The expression level of membrane molecules on the cell surface was assessed by flow cytofluorometry (BD FACSCanto II analyzer) using monoclonal antibodies labeled with fluorescein isothiocyanate (anti-CD80, anti- CD123) or phycoerythrin (anti-HLA-DR, anti-CD86) (Caltag, USA).

The cytokine concentration in the culture media was determined by ELISA using the proper test kits (OAO Cytokine, St. Petersburg, Russia).

Intracellular cytokines were determined in mononuclear cells activated by a mixture of 4-phorbol 12-myristate 13-acetate (PMA) and ionomycin (iono) in the presence of BD GolgiStop (Becton Dickinson, USA) and permeabilized using the BD Cytofix/Cetoperm Fixation/ Permeabilization Kit on a flow cytometer using labeled anti-cytokine monoclonal antibodies [51].

The expression levels of the transcription factor genes (NF-κB, GATA-3, Tbet, FOXP3 and RORc) were determined by a real-time reverse-transcription polymerase chain reaction. The TaqMan One-Step RT - PCR Master Mix Reagents Kit and TaqMan Gene Expression Assays (Applied Biosystems, USA) were used [52]. The mRN A expression level was determined with respect to the expression mRN A level in the housekeeping gene of β2-microglobulin (B2M) according to the formula:

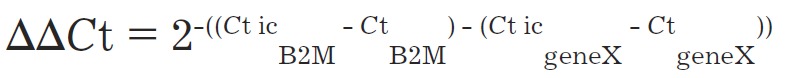



where *C*t is the threshold cycle determined in the exponential portion of the DNA accumulation curve and IC is the internal control.

The results were statistically processed using nonparametrical methods for data analysis. The indices were represented as Me (L–H), where Me is the median value and L and H are the lower and higher quartiles, respectively. The Mann–Witney U-test was used to compare the indicators.

## RESULTS AND DISCUSSION

Neolact was found to activate innate immunity: at concentrations of 10 and 100 μg/ml, Neolact significantly boosted the secretion of IL-1β by human blood monocytes, while having no effect on TN Fα secretion.

**Fig. 1:**
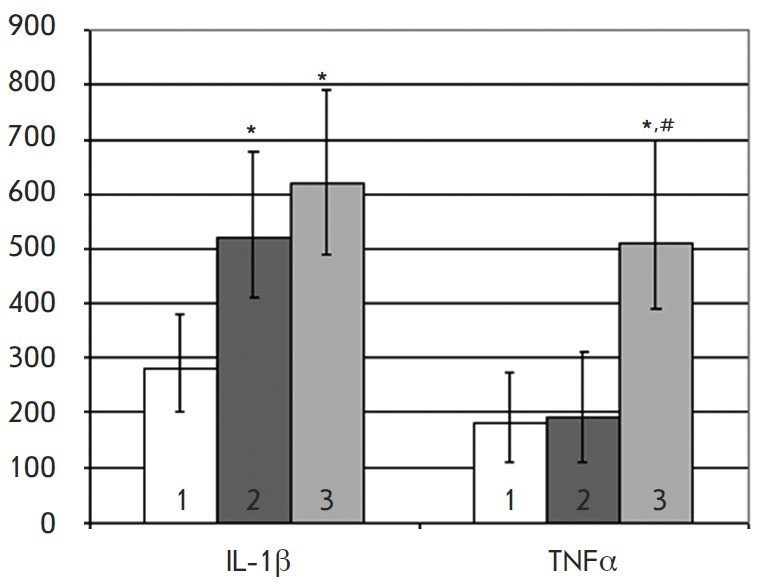
The effect of Neolact (*2*) and Neolact enriched in iron (*3*) on the monocyte secretion of cytokines IL-1β and TNFα. *1 *–cytokine secretion level in the control. Y axis: cytokine concentrations (pg/ml) in the culture medium of monocytes. Medians *p  < 0.05 regarding the control are presented; # – the same regarding Neolact. The concentration of Neolact and Neolact enriched in iron is 10 μg/ml

**Fig. 2:**
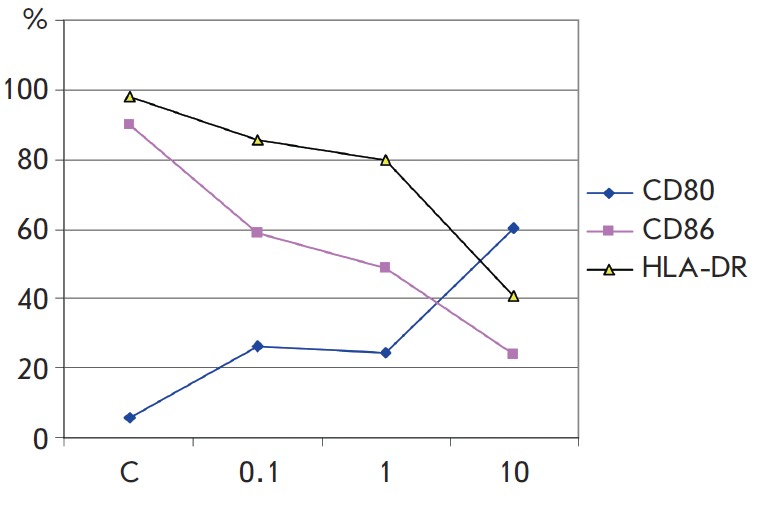
The effect of Neolact on the expression of the costimulatory molecules HLA-DR, CD80, and CD86 by HTSC. IL10 dendritic cells. The mean values of three experimental runs are shown. X axis – Neolact concentration, μg/ml, Y axis – percentage of cells carrying a marker. C – original expression of costimulatory molecules without adding Neolact

The enrichment of Neolact in iron ions induced the ability to boost TN Fα secretion (*Fig. 1*). Thus, the proinflammatory activity of Neolact was limited by an increase in IL-1β secretion by blood monocytes, while its enrichment in iron ions activated the innate immunity and enhanced the manifestation of pro-inflammatory effects to a significant extent.

**Fig. 3:**
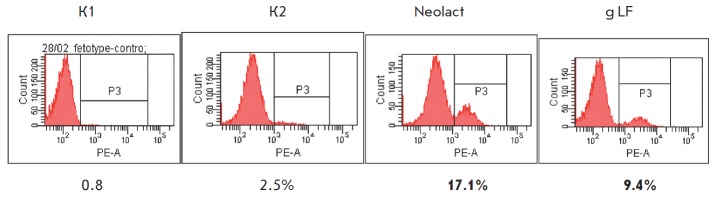
The effect of Neolact and goat lactoferrin on the expression of CD123 molecules on HTSC.IL10 dendritic cells in one-day-old cultures. Histograms of two-color staining with monoclonal antibodies. K1 – without anti-CD123-PE staining; K2 – without incubation with Neolact. Values that differ from the control at least twofold are shown in bold. The concentrations of Neolact and goat lactoferrin were 10 μg /ml

Figure 2 shows the effect of Neolact on the expression of the membrane molecules that play a crucial role in antigene presentation, which was determined for HTSC.IL-10 dendritic cells. Neolact at three tested doses significantly reduced the number of cells expressing major histocompatibility complex class II (HLA-DR) molecules and the costimulatory molecule CD86, which were originally present in almost all cells in this cell line, and increased the number of cells carrying another costimulatory molecule (CD80), which was originally contained in a small number of cells in this cell line. Neolact actually induced the replacement of costimulatory molecules on the surface of dendritic cells. Meanwhile, the density of HLA-DR molecules on each individual cell increased under the action of Neolact. These effects were eliminated by enriching the drug in iron ions. The decrease in the percentage of dendritic cells carrying HLA-DR molecules can be considered as evidence of the fact that Neolact limits the antigen-presenting ability of a dendritic cell population. Neolact causes no quantitative changes in T-cell activation dependent on the expression of costimulatory molecules, since attenuation of the expression of one costimulatory molecule is accompanied by the enhancement of the expression of another molecule performing the same function. Meanwhile, Neolact exhibits the activity of a dendritic cell differentiation factor: this can be seen from the expression of the marker for plasmacytoid dendritic cells (CD123), which is an IL-3 receptor (Fig. 3). The induction of CD123 expression, which can be interpreted as a sign of the conversion of the dendritic cell phenotype from myeloid to plasmacytoid [53], determines the Th2-type immune response and attenuates the more aggressive response of T cells (Th1 and Th17) that causes immune inflammation. It should be mentioned that the differentiating ability of gLF is pronounced to a much lesser extent (Fig. 3).

The choice of the differentiation direction of Thelper cells eventually determines the direction of the immune response, whether it is pro- or anti-inflammatory, the ability to promote the development of various forms of immune pathology, etc. Th1- and Th17 cells can be conventionally classified as pro-inflammatory cells, while Th2 and Treg can be classified as anti-inflammatory ones. Of note, Th2 cells are typically regarded as proallergic cells. The differentiation direction and stabilization of the cell phenotype is determined by the expression of the GATA-3 (for Th2 cells), Tbet (for Th1), RORc (for Th17), and FOXP3 (for Treg) transcription factors, which are encoded by the* GATA3, TBX21, RORC, *and *FOXP3 *genes, respectively. In this context, the range of expression of the specified genes by blood T cells significantly predetermines the hereditary or induced tendency of the organism to develop certain types of the immune response and various forms of immune pathology.

**Table:** Effect of Neolact on the expression of the genes of the transcription factors that control the differentiation of CD4+ T-lymphocytes

Neolact, μg/ml	Transcription factor genes			
	GATA3	TBX21	RORC	FOXP3
Nonactivated lymphocytes				
0 (control)	0.718 (0.527–0.974)	0.010 (0.005–0.018)	0.260 0.199–0.292)	0.569 (0.306–0.818)
1.0	1.173* (0.815–1.690)	0.014 (0.002–0.016)	0.266 (0.159–0.272)	0.834* (0.811–1.120)
10.0	0.727 (0.481–2.587)	0.018 (0.001–0.028)	0.172 (0.043–0.409)	0.767 (0.246–0.774)
Phytohemagglutinin-activated lymphocytes				
0 (control)	0.613 (0.483–0.894)	0.010 (0.005–0.017)	0.649 (0.433–1.013)	0.805 (0.047–1.101)
1.0	1.228* (0.705–1.815)	0.014 (0.007–0.018)	0.487 (0.399–0.802)	1.018 (0.759–2.446)
10.0	0.675 (0.399–0.807)	0.011 (0.008–0.013)	0.743 (0.483–1.576)	0.678 (0.361–1.069)

*p < 0.05. Note. Medians are presented (the lower and upper quartiles are shown in brackets).

The effect of Neolact on the development of various T-helper cells was assessed according to their effect on the expression of the transcription factor genes that regulate CD4+ T-cell differentiation (Table). Neolact and its iron-enriched derivative at concentrations as low as 1 μg/ml enhanced the expression of the *GATA3* gene responsible for the development of Th2 cells, antiparasite protection, and pro-allergic orientation of the immune processes. The effect of Neolact could be seen for both resting and activated T cells. No significant effect on the expression of the “pro-inflammatory” genes* TBX21 *(encodes the Tbet factor of Th1 cells) and *RORC* (encodes the RORc factor of Th17 cells) have been detected. Neolact enhanced the expression of the *FOXP3* gene responsible for the development of regulatory T cells, which limit the intensity and duration of the immune response. Neolact does not induce expression in the dendritic cells of the gene of the IL-12 beta chain, which is responsible for Th1 cell differentiation.

Thus, Neolact exhibited no ability to stimulate the expression of the factors contributing to the development of immune inflammation in this series of tests. Instead, it had the opposite effect as it stimulated the expression of the genes responsible for the development of Th2 and Treg cells.

**Fig. 4:**
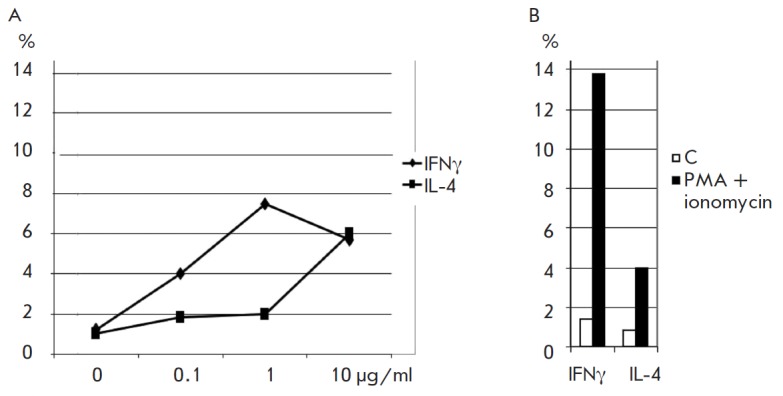
(A) The effect of Neolact on the induction of the T cells forming IFNγ and IL-4 (*n*=3). (B) positive control of the specified cytokines by T cells under optimal stimulation with PMA/ionomycin (100 nM/2 μM, respectively). X-axis – rhLF concentration (*A*); Y-axis – the percentage of cells producing the specified cytokines (*A, B*)

The assessment of the effect of Neolact on the differentiation of Th1 and Th2 cells (Th1 and Th2 cells were determined according to the number of cells producing their key cytokines, IFNγ and IL-4, respectively) has demonstrated that Neolact enhances the secretion of both cytokines. Neolact at a concentration of 1 μg/ml increases IFNγ secretion to a greater extent than IL-4 secretion. The secretion of both cytokines becomes identical at a Neolact concentration of 10 μg/ml (Fig. 4). However, the number of cells producing IFNγ remains much lower event at a Neolact concentration of 1 μg/ml than it is when cells are activated with a PMA–ionomycin mixture. The number of IL-4-producing cells is higher than this level. In other words, stimulation of Th2 cells with Neolact corresponds to the physiological level of their involvement in the immune response, whereas stimulation of Th1 cells remains below this level, which is consistent with data obtained by assessing the effect of rhLF on the expression of the genes of the transcription factor regulating the differentiation of T-helper cell subtypes.

## CONCLUSIONS

Summarizing the results of the assessment of the effect of Neolact on certain manifestations of the immune system activity, one can draw a conclusion that the agent exhibits immunotropic activity and that its effect is associated with either inhibition of immune processes or their development via the Th2-dependent pathway to a certain extent. Meanwhile, according to its effect on the formation of the cells producing IFNγ- and IL-4, the agent does not cause a strong polarization of the immune response, which could have resulted in the development of allergic or autoimmune processes. Neolact enriched in iron ions is characterized by an enhanced pro-inflammatory activity and lacks a number of the effects that are typical of original Neolact.

## References

[1] Farnaud S., Evans R.W. (2003). Mol. Immunol..

[2] Brock J.H. (2012). Biochem. Cell Biol..

[3] Sallmann F.R., Baveye-Descamps S., Pattus F., Stivense K.J. (1999). J. Biol. Chem..

[4] Van Hooijdonk A.C., Kusstndrager K.D., Steijns J.M. (2000). Br. J. Nutr..

[5] Ikeda M., Nozaki A., Sugiyama K., Shiita J., Sato T. (2000). Virus Res..

[6] Pietrantoni A., Ammendolia M.G., Superti F. (2012). Biochem. Cell Biol..

[7] Andersson Y., Lindquist S., Lagerqvist C., Hernell O. (2000). Early. Hum. Dev..

[8] Giels S., Czuprynski C. (2002). Microb. Pathog..

[9] Kruzel M.L., Zimecki M. (2002). Arch. Immunol. Ther..

[10] Leitch E.C., Willcox M.D. (2001). Int. J. Antimicrob. Agents..

[11] Naidu A.S., Arnold R.R. (1994). Diagn. Microbiol. Infect. Dis..

[12] Fowler C.E., Soothill J.S., Oakes L. (1997). J. Antimicrob. Chemother..

[13] Reiter B. (1978). Ann. Rech. Vet..

[14] Petschow B.M., Talbott R.D., Batema R.P. (1999). J. Med. Microbiol..

[15] Kim W.S., Ohashi M., Tanaka T., Nozaki A., Sugiyama K. (2004). Biometals..

[16] Yamauchi K., Wakabayashi H., Shin K., Takase M. (2006). Biochem. Cell Biol..

[17] Tomita M., Wakabayashi H., Shin K., Kuwata H., Yip T.T., Yamauchi K., Teraguchi S., Hayasawa H. (2009). Biochimie..

[18] Mulder A.M., Connellan P.A., Oliver C.J., Morris C.A., Stevenson L.M. (2008). Nutr. Res..

[19] Kulics J., Kijstra A. (1986-1987). Immunol. Lett..

[20] Furlong S.J., Mader J.S., Hoskin D.W. (2010). Exp. Mol. Pathol..

[21] Mader J.S., Salsman J., Conrad D.M., Hoskin D.W. (2005). Mol. Cancer Ther..

[22] Bezault J., Bhimani R., Wiprovnick J. (1994). Cancer Research.

[23] Murphy M.E., Kariwa H., Mizutani T., Yoshimatsu K., Arikawa J., Takashima I. (2000). Arch. Virol..

[24] Buharin O.V., Valishev A.V., Valisheva I.V. (2011). Uspehi_ Sovremennoy_Biologii (Biology Bulletin Reviews)..

[25] Lonnerdal B., Iyer S. (1995). Annu. Rev. Nutr..

[26] Spadaro M., Caorsi C., Ceruti P., Varadhachary A., Forni G., Pericle F., Giovarelli M. (2008). FASEB J..

[27] Nemtsova E.R., Ivanova L.M., Yakubovskaya R.I. (1995). Biomedical chemistry..

[28] Edeleva N.V., Sergeeva T.V., Nemtsova E.R., Tsherbitskaya I. Ya., Yakubovskaya R.I., Osipova N.A. (2001). Anesteziology and reanimatology..

[29] Nemtsova E.R., Edeleva N.V., Osipova N.A., Yakubovskaya R.I., Chissov V.I. (2006). Russian oncology..

[30] Edeleva N.V., Nemtsova E.R., Yakubovskaya R.I., Osipova N.A. (2005). Russian oncology..

[31] Chissov V.I., Yakubovskaya R.I., Boyko A.V. Pat..

[32] Spik G., Burnet B., Mazurier-Dehaine C., Fontaine G., Montreuil J. (1982). Acta Paediatr. Scand..

[33] Cirioni O., Giacometti A., Barchiesi F., Scalise G. (2000). J. Antimicrob. Chemother..

[34] Valenti P., Berlutti F., Conte M.P., Longhi C., Seganti L. (2004). J. Clin. Gastroenterol..

[35] Magnuson J.S., Henry J.F., Yip T.T., Hutchens T.W. (1990). Pediatr. Res..

[36] Kawakami H., Londderdal B. (1991). Am. J. Physiol..

[37] Suzuki Y.A., Shin K., Lonnerdal B. (2001). Biochemistry..

[38] Baker E.N., Baker H.M. (2005). Cell Mol. Life Sci..

[39] Suzuki Y.A., Lopez V., Lonnerdal B. (2005). Cell. Mol. Life Sci..

[40] Bellamy W., Takase M., Wakabayashi H., Kawase K., Tomita M. (1992). J. Appl. Bacteriol..

[41] Odell E.W., Sarra R., Foxworthy M., Chapple D.S., Evans R.W. (1996). FEBS Lett..

[42] Conesa C., Calvo M., Sánchez L. (2010). Biotechnol. Adv..

[43] Lönnerdal B. (2002). J. Am. Coll. Nutr..

[44] Andersen J.H. (2004). Curr. Opin. Mol. Ther..

[45] Van Berkel P.H., Welling M.M., Geerts M., van Veen H.A., Ravensbergen B., Salaheddine M., Pauwels E.K., Pieper F., Nuijens J.H., Nibbering P.H. (2002). Nat. Biotechnol..

[46] Zhang J., Li L., Cai Y., Xu X., Chen J., Wu Y., Yu H., Yu G., Liu S., Zhang A. (2008). Protein. Expr. Purif.

[47] Goldman I.L., Georgieva S.G., Gurskiy Y.G., Krasnov A.N., Deykin A.V., Popov A.N., Ermolkevich T.G., Budzevich A.I., Chernousov A.D., Sadchikova E.R. (2012). Biochem. Cell Biol..

[48] Sokolov A.V., Pulina M.O., Kristiyan A.V., Zaharova E.T., Runova O.L., Vasilev V.B., Gurskiy Y.G., Minashkin M.M., Krasnov A.N., Kadulin C.G. (2006). DAS..

[49] Goldman I., Chernousov A., Sadchikova E. (2010). Recent Adv. Clin. Med..

[50] Sharova N.I., Litvina M.M., Yarilin A.A. (2010). Russian Immunology..

[51] Jung T., Schauer U., Heusser C., Neumann C., Rieger C. (1993). J. Immunol. Meth..

[52] Donetskova A.D., Nikonova M.F., Yarilin A.A. (2011). Russian Immunology..

[53] Schmitt N., Cumont M.C., Nugeyre M.T., Hurtrel B., Barré-Sinoussi F., Scott-Algara D., Israël N. (2007). Immunobiology..

